# A Facile Surface Reconstruction Mechanism toward Better Electrochemical Performance of Li_4_Ti_5_O_12_ in Lithium‐Ion Battery

**DOI:** 10.1002/advs.201700205

**Published:** 2017-07-10

**Authors:** Kun Qian, Linkai Tang, Marnix Wagemaker, Yan‐Bing He, Dongqing Liu, Hai Li, Ruiying Shi, Baohua Li, Feiyu Kang

**Affiliations:** ^1^ Nano Energy Materials Laboratory (NEM) Tsinghua‐Berkeley Shenzhen Institute (TBSI) Tsinghua University Shenzhen 518055 P. R. China; ^2^ Laboratory of Advanced Materials School of Materials Science and Engineeing Tsinghua University Beijing 100086 P. R. China; ^3^ Engineering Laboratory for the Next Generation Power and Energy Storage Batteries Graduate School at Shenzhen Tsinghua University Shenzhen 518055 P. R. China; ^4^ Department of Radiation Science and Technology Delft University of Technology Mekelweg 15 Delft 2629JB The Netherlands

**Keywords:** hydrothermal method, Li_4_Ti_5_O_12_, lithium‐ion batteries, Na_2_S, surface modifications

## Abstract

Through a facile sodium sulfide (Na_2_S)‐assisted hydrothermal treatment, clean and nondefective surfaces are constructed on micrometer‐sized Li_4_Ti_5_O_12_ particles. The remarkable improvement of surface quality shows a higher first cycle Coulombic efficiency (≈95%), a significantly enhanced cycling performance, and a better rate capability in electrochemical measurements. A combined study of Raman spectroscopy and inductive coupled plasma emission spectroscopy reveals that the evolution of Li_4_Ti_5_O_12_ surface in a water‐based hydrothermal environment is a hydrolysis–recrystallization process, which can introduce a new phase of anatase‐TiO_2_. While, with a small amount of Na_2_S (0.004 mol L^−1^ at least), the spinel‐Li_4_Ti_5_O_12_ phase is maintained without a second phase. During this process, the alkaline environment created by Na_2_S and the surface adsorption of the sulfur‐containing group (HS^−^ or S^2−^) can suppress the recrystallization of anatase‐TiO_2_ and renew the particle surfaces. This finding gives a better understanding of the surface–property relationship on Li_4_Ti_5_O_12_ and guidance on preparation and modification of electrode material other than coating or doping.

## 1 Introduction

As one of the most promising solutions for energy storage and utilization, lithium‐ion batteries (LIBs) have been attracting tremendous interests on a world scale. Over the past few decades, LIBs with carbonaceous anodes have dominated the portable power market.^1^, ^2^ However, this commercial success still cannot extend to the area of large‐scale energy storage and high power applications at present, in which high safety, long cycle life, and high power density are highly demanded.^3^, ^4^ Among the complex causes of the existing defects of commercial LIBs, carbonaceous anodes play a significant role. First of all, the Li‐insertion voltage of commercial graphite anode is at ≈100 mV versus Li^+^/Li, which is very close to the redox potential of lithium metal.^5^, ^6^ Thus, under the condition of high‐power charge rates, lithium plating is prone to occur on the surface of the anode due to an extensive polarization of carbon materials, then potentially triggering thermal runaway when some lithium dendrites penetrate into the separator. Also, the reduction of electrolyte and formation of solid electrolyte interphase (SEI) layer on the carbon anode, which usually occurs around 1.0 V versus Li^+^/Li, can result in a considerable consumption of electrolyte and cyclable lithium, showing reduced cycle and calendar life.^7^, ^8^

To develop advanced LIBs that will meet the requirements of large‐scale energy storage, a series of materials have been investigated as alternative anode materials. Among the candidates, spinel Li_4_Ti_5_O_12_ (LTO) is attractive to scale up and make high performance and cheap static energy storage viable. Unlike the carbonaceous anode, LTO has a higher Li‐insertion voltage (≈1.5 V vs Li^+^/Li),^9^, ^10^ which can avoid the formation and breakdown of the SEI layer and reduce the loss of cyclable lithium and electrolyte.^11^, ^12^ During charging and discharging, the spinel LTO lattice structure displays practically no volume change. This so‐called zero‐strain property is responsible for its excellent reversibility upon cycling, making it potentially an ultralong cycle life, and high safety negative electrode material compared to the graphite anode.^13^, ^14^, ^15^

However, the commercially available micrometer‐sized LTO particles have not achieved the outstanding performance that may be expected from the promising material properties. The low intrinsic electronic and ionic conductivity is considered as the main reason. Two main strategies were proposed to improve the conductivity. One strategy is to fabricate a nano‐ or porous structure, which enlarges the specific surface area (over 200 m^2^ g^−1^), and shortens the ion and electron diffusion lengths.^16^ The other strategy is the construction of a conductive network by adding conducting agent (such as carbon nanotubes, graphene, and other conductive carbon),^17^, ^18^, ^19^, ^20^ or doping,^11^, ^21^ or coating.^8^, ^22^ Although these approaches have been reported effective in terms of excellent high rate capabilities, the nanosizing compromises the tap density and colloidal stability, and the complicated preparation procedures are accompanied by high costs, making this strategy difficult to apply in practice.^5^

Here, we propose a different strategy to improve the LTO performance, focusing on the improvement of the quality of the LTO surface and staying within the boundary condition of the low cost and facile modification methods based on the available LIB manufacturing technology. Large‐scale production of LTO introduces surface defects, for example, an amorphous layer or the presence of impurity ions. In this work, for the first time, we describe a simple surface modification mechanism to prepare LTO particles with pure, clean, and nondefective surfaces through simple hydrothermal treatment with aqueous solution of sodium sulfide (Na_2_S). The modified LTO particles possess a higher first cycle Coulombic efficiency (≈95%), a significantly enhanced cycling performance, and a better rate capability, making this approach effective and promising for application in LIB technology. The modification process was systematically investigated and based on our results we propose the following mechanism: Under the hydrothermal environment of water, the LTO particles experienced a hydrolysis‐recrystallization process, which introduces anatase‐TiO_2_ at the LTO surface. Benefiting from the alkaline environment created by Na_2_S and the surface adsorption of the sulfur‐containing group (HS^−^ or S^2−^), the hydrolysis of LTO is reduced. At the same time, the recrystallization of anatase‐TiO_2_ is suppressed entirely. Thus, the LTO phase is protected while the amorphous phase and impurity ions are removed by the hydrolysis. The surface reconstruction mechanism and the corresponding surface structure–electrochemical performance relationship were characterized by Raman, inductive coupled plasma emission spectrometer (ICP) and cyclic voltammetry (CV) tests. This finding gives guidance to electrode material preparation and modification strategies. Furthermore, the proposed approach is facile and easy to scale up and can be combined with other treatments, such as coating or doping, for improving the performance of micro‐ or nanosized LTO electrode materials.

## 2 Results and Discussion

**Figure 1:**
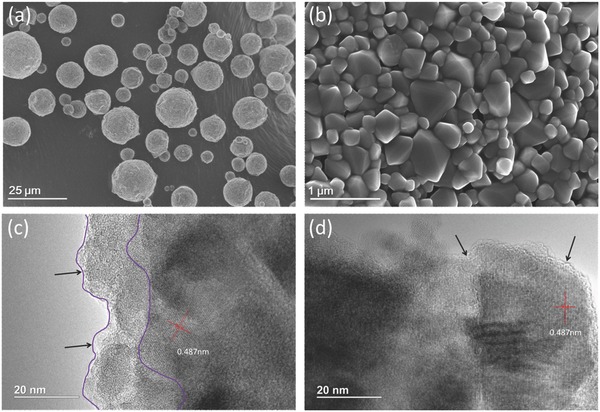
Morphology and structure of the spherical Li_4_Ti_5_O_12_ materials: a) SEM image of the pristine sample; b) magnified SEM image of the pristine sample; HR‐TEM image of c) pristine sample and d) modified sample (0.008 mol L^−1^ Na_2_S aqueous solution, 180 °C, 8 h).

The LTO particles show a suitable morphology for battery application,^23^ namely, micrometer‐sized (≈12–20 µm) secondary particles (**Figure** **1**a) composed of ≈100–500 nm primary particles (Figure 1b). The spherical particles are easy to disperse and allow obtaining a high tap density (1.55 g cm^−3^), which is a prerequisite for a large volumetric energy density. After modification with Na_2_S solution, the size and morphology of LTO particles do not change noticeable. However, through high‐resolution transmission electron microscopy (HR‐TEM), it is observed that the very thin (about 10 nm) outermost amorphous layer on the pristine LTO surface was totally removed (Figure 1c,d), displaying a clear lattice fringe (Figure 1d). The average lattice spacing is 0.487 nm corresponding to the LTO (111) plane, indicating the LTO crystal structure is well maintained after 8 h hydrothermal treatment with Na_2_S. The amorphous layer on the pristine LTO surface is probably generated during solid‐phase sintering, in which some impurity elements aggregated at the surface of the LTO particles, creating defects, and preventing the ordered arrangement of the Li, Ti, and O ions.^15^ The significant signal of Cl 2s and Cl 2p X‐ray photoelectron spectroscopy (XPS) confirmed the presence of this impurity element in the amorphous layer of pristine LTO (Figure S1, Supporting Information). Disappearance of this impurity XPS signal in the modified LTO provided additional evidence on the highly improved quality of LTO surface.

**Figure 2:**
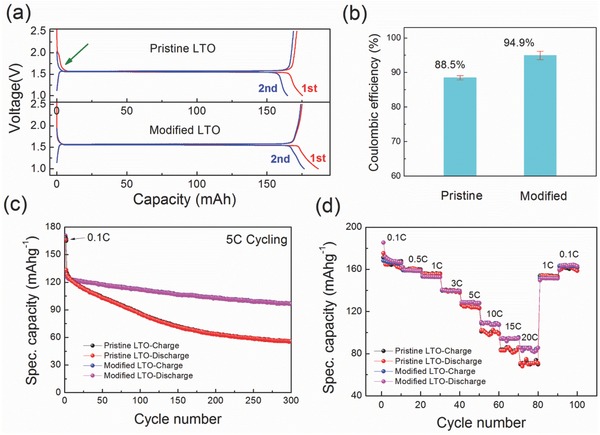
Electrochemical performance of the pristine and modified Li_4_Ti_5_O_12_ materials: a) charge–discharge profile of first two cycles at 0.1 C (1 C = 175 mA g^−1^) in a voltage range from 1.0 to 2.5 V; b) the first cycle Coulombic efficiency of pristine and modified samples; c) cycling performance at 5 C current and d) rate capability from 0.1 to 20 C.

Coin‐type cells were assembled to evaluate the electrochemical properties of pristine LTO and modified LTO. **Figure** **2**a shows the first and second charge–discharge profiles at 0.1 C rate (C = 175 mAh g^−1^). The results show that the surface modification significantly improved the reversibility of lithium intercalation and deintercalation. This is supported by comparing the voltage during the initial stages of Li‐ion insertion between the first and the second cycle as indicated in Figure 2a. Clearly, there is a difference in voltage between 1.5 and 2.0 V for the pristine LTO, whereas it is absent for the surface‐modified LTO. For anode materials, a difference in voltage usually indicates the occurrence of undesirable side reactions, associated with irreversible capacity loss. For example, this phenomenon has often been observed in graphite anodes at ≈0.8 V (vs Li/Li^+^) during the initial discharge, in which the electrolyte decomposed on the graphite surface and then formed a SEI, consuming cyclable lithium, lowering the Coulombic efficiency, and making obvious voltage deviation.^24^, ^25^, ^26^ However, for LTO anodes, the Li^+^ insertion–extraction process occurs at 1.55 V (vs Li/Li^+^), far from the decomposition voltage of electrolyte and it is generally accepted that LTO surface cannot form a SEI film.^27^ Thus, the origin of the side reaction most likely comes from the interaction between amorphous layer with the electrolyte. The increase of the average first Coulombic efficiency from 88.5% (pristine) to 94.9% (modified), based on 12 coin‐type cells for each sample (Figure 2b), also strongly suggests that the impure and amorphous surface of pristine LTO mainly leads to the consumption of cyclable lithium and electrolyte. Furthermore, the side reactions not only cause evident Coulombic efficiency loss during the first one or two cycles, but also severely impaired the cycle life of LTO. Figure 2c compares the cycle performance of pristine and modified LTO under 5 C‐rate current. After 300 discharge–charge cycles, the modified LTO with clean surfaces delivers a specific capacity retention as high as 75%, whereas the pristine sample only keeps 44% of the initial capacity. Figure 1d displays the rate capabilities cycled at current rates from 1 to 20 C. For the currents smaller than 5 C, there is no difference in the specific capacity between the pristine and modified LTO. However, when the cycling rate increases to 10 C and larger, the modified LTO shows better capacity retention. It suggests that the 10 nm amorphous layer, to some degree, forms a diffusion barrier for Li^+^ between the electrolyte and the spinel LTO phase, which hinders the charge‐transfer process, which apparently becomes rate limiting at large currents. Although the rate performance does not achieve that of carbon‐coated LTO, nano/porous LTO, or other samples based on methods aiming at improving the conductivity,^28^, ^29^, ^30^, ^31^, ^32^, ^33^ these results demonstrate the importance of the surface quality for the performance of electrode materials, giving guidance to design and produce high‐performance materials.

**Figure 3:**
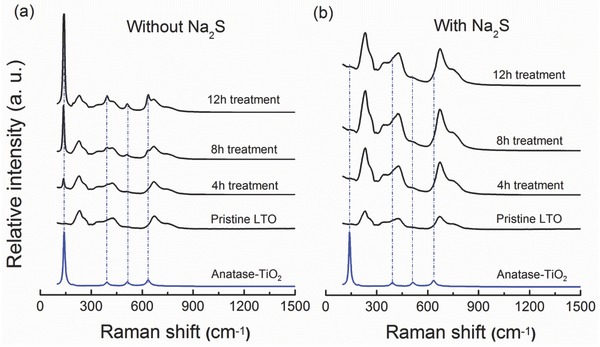
Raman spectra of hydrothermal treated Li_4_Ti_5_O_12_ a) with water and b) with 0.008 mol L^−1^ Na_2_S aqueous solution.

In order to find out the surface reconstruction mechanism during a simple hydrothermal treatment, Raman spectroscopy, mainly sensitive to particle surface and near‐surface region, was employed to measure a series of LTO samples. **Figure** **3**a shows the Raman spectroscopy results of the pristine and the hydrothermally treated samples with water (0 mol L^−1^ Na_2_S) for 4, 8, and 12 h, in turn (black color). The Raman spectrum of the initial sample is fully assigned to the F2g mode of bending vibration δ(Ti–O), can be taken as a characteristic Raman fingerprint of LTO. After 4 h treatment, another group Raman bands appeared and the intensity of these bands increased with the extension of hydrothermal time. According to the theoretical calculation and the Raman result of standard sample of anatase‐TiO_2_ (blue color in Figure 3a), all the emerging Raman bands centered at 141, 392, 512, and 637 cm^−1^ are attributed to anatase‐TiO_2_. Powder X‐ray diffraction results also support the existence of anatase‐TiO_2_ (Figure S2, Supporting Information). Also, the most intense Raman band at 141 cm^−1^ corresponds to a characteristic Raman indicator of anatase‐TiO_2_. Herein, we define the intensity ratio of Raman bands at 141 and 231 cm^−1^ (*I*_141_/*I*_231_ or A/L) as the indicator of anatase‐TiO_2_ content. In other words, the higher the value of A/L, the higher the content of anatase‐TiO_2_. The Ti‐O bonds in anatase‐TiO_2_ possess large polarity which tends to react with trace water in electrolyte and form hydroxyl groups on the surface. The hydroxyl groups are Lewis‐acid sites which are considered to be able to initiate the decomposition of electrolyte solvent. Due to this high catalytic activity, anatase‐TiO_2_ is considered as an undesired phase and potentially triggers severe side reactions and gassing.^34^, ^35^ Interestingly, for the hydrothermal treated LTO with 0.008 mol L^−1^ Na_2_S aqueous solution, the A/L value is always zero from 4 to 12 h, indicating that Na_2_S prevents the formation of anatase‐TiO_2_ phase on the LTO surface. Consistently, no X‐ray diffraction peaks of anatase‐TiO_2_ were observed (Figure S2, Supporting Information). To verify this result, high resolution of synchrotron X‐ray was employed for the sample after 12 h treatment. From the synchrotron data, no anatase‐TiO_2_ phase can be found indicting the total inhibition of TiO_2_ phase (Figure S3, Supporting Information). Furthermore, compared with the Raman signal of the pristine sample, a significant enhancement was observed in samples after Na_2_S‐assisted hydrothermal treatments (Figure 3b), actively supporting the removal of amorphous layer observed by HR‐TEM.

**Figure 4:**
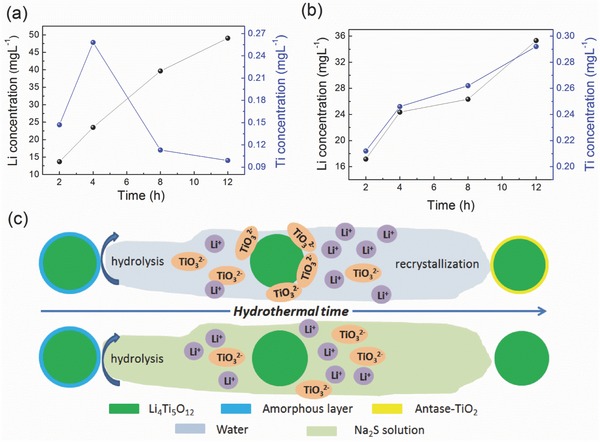
The concentration evolution of soluble Li and Ti (data acquired from Inductive Coupled Plasma Emission Spectra) during the hydrothermal treatment process: a) with water and b) with 0.008 mol L^−1^ Na_2_S aqueous solution. c) Surface modification mechanisms of Li_4_Ti_5_O_12_ under hydrothermal environment of water and Na_2_S solution.

Through a series of analogous experiments performed with initial LTO (5 g) and different concentrations of Na_2_S (10 levels from 0 to 0.02 mol L^−1^) for 8 h (Figure S4 and Table S1, Supporting Information), it is suggested that the anatase‐TiO_2_ phase can be inhibited when the Na_2_S concentration exceeds 0.004 mol L^−1^ resulting in a high‐quality LTO surface. **Figure** **4**a displays the concentration of soluble lithium and titanium in the solution during hydrothermal treatment (0 mol L^−1^ Na_2_S) as a function of time. The data suggest that the dissolved amount of lithium always increases with time, while the soluble Ti first reaches a peak concentration at 4 h (0.26 mg L^−1^) after which it decreases to a relatively low level. Thus, combined with the Raman results, this infers that the surface evolution without Na_2_S includes two stages. In the first stage, the outmost layer of Li_4_Ti_5_O_12_ begins to hydrolyze, during which the amount of soluble Li and Ti experiences an increase. The hydrolytic process is shown in Equations (1) and (2). Li_4_Ti_5_O_12_ first transforms to TiO(OH)_2_ and the Li^+^ dissolves, subsequently some TiO(OH)_2_ transforms to dissolved TiO_3_^2−^ in water. In the second the stage, Li_4_Ti_5_O_12_ continues releasing the Li^+^ from the lattice, while TiO(OH)_2_ no longer hydrolyzes but transforms to anatase‐TiO_2_ (Equation (3)). Additionally, some dissolved TiO_3_^2−^ may be absorbed again to the particle surface which then recrystallized to anatase‐TiO_2_ (Equation (4)). This explains why in the second stage the concentration of Li continues increasing while the concentration of Ti drops to a relatively small value.

(1) $${\mathrm{Li}}_{4}{\mathrm{Ti}}_{5}O_{12}\left(s\right)+7H_{2}O=5\mathrm{TiO}{\left(\mathrm{OH}\right)}_{2}\left(s\right)+4{\mathrm{Li}}^{+}+4{\mathrm{OH}}^{-}$$

(2) $$\mathrm{TiO}{\left(\mathrm{OH}\right)}_{2}\left(s\right)+2{\mathrm{OH}}^{-}={\mathrm{TiO}}_{3}{}^{\;\;2-}+2H_{2}O$$

(3) $$\mathrm{TiO}{\left(\mathrm{OH}\right)}_{2}={\mathrm{TiO}}_{2}+H_{2}O$$

(4) $${\mathrm{TiO}}_{3}{}^{\;\;2-}+H_{2}O={\mathrm{TiO}}_{2}+{\mathrm{2OH}}^{-}$$

This process can be described as the hydrolysis‐recrystallization process (Figure 4c). When some Na_2_S was added to the solution, the hydrolysis‐recrystallization process was disturbed. It is directly proved by the ICP data (Figure 4b, 0.008 mol L^−1^ Na_2_S) that both of the Li and Ti concentration increase, not reaching a maximum during the hydrothermal process. Based on our results, we propose the following surface reconstruction mechanism of LTO, depicted in Figure 4c. The impact of the presence of Na_2_S manifests itself in two aspects. First, the alkaline environment created by Na_2_S may, to some extent, weaken the hydrolysis of Li_4_Ti_5_O_12_.^36^ For this reason, the Li concentration after 12 h treatment with Na_2_S is only about 70% of that without Na_2_S (Figure 4a,b). Second, some S^2−^ or HS^−^ ions may be adsorbed on the particle surface or replace the position of O^2−^ on the surface region, enhancing the steric hindrance, avoiding the absorption of TiO_3_^2−^, and making the recrystallization process impossible (Figure S5, Supporting Information). The consequence is that in the presence of Na_2_S only the hydrolysis occurs which completely removes the amorphous layer including the impurity elements resulting in a pure, clean, and nondefective surface.

**Figure 5:**
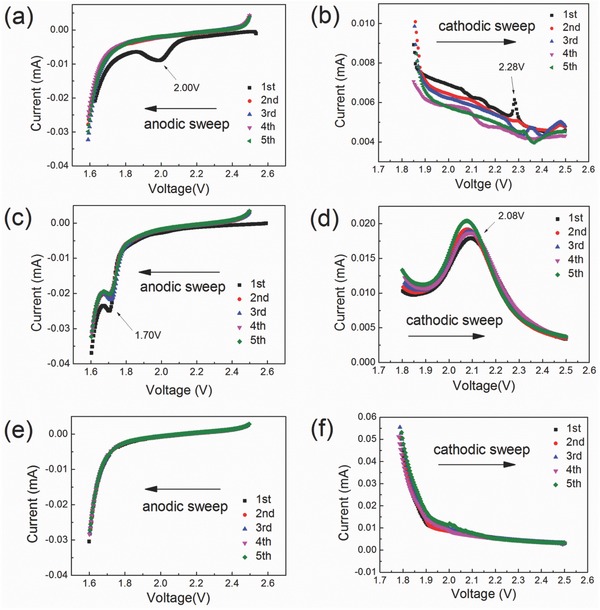
Selected parts of the cyclic voltammetry curves at a scan rate of 0.1 mV s^−1^: a,b) pristine Li_4_Ti_5_O_12_; c,d) hydrothermal treated sample with water; e,f) hydrothermal treated sample with 0.008 mol L^−1^ Na_2_S.

CV measurements were carried out to investigate the relationship of the surface structure with the electrochemical behavior. Figure S6 (Supporting Information) exhibits the typical CV curve of LTO electrode at a scan rate of 0.1 mV s^−1^ for five cycles. A pair of sharp and intense redox current peaks is observed at about 1.66 and 1.49 V on all the tested samples including the pristine LTO, the hydrothermal treated LTO with water, and with 0.008 mol L^−1^ Na_2_S aqueous solution. The peaks were due to the Li^+^ insertion and deinsertion in LTO.^37^ Further analysis of the three samples identified that some subtle distinctions are hidden in the CV curves. **Figure** **5** shows the detailed difference during the selected part of the CV curves, namely, between 2.5 and 1.6 V during the anodic sweep and between 1.8 and 2.5 V during the cathodic sweep. Figure 5a,b displays the CV details of pristine LTO, showing a distinct anodic current peak at 2.0 V during the first lithium insertion process and an almost invisible cathodic current peak in corresponding delithiation process. This is in agreement with our inference that some irreversible reaction occurs on the amorphous layer. Moreover, the nonoverlapping CV curves in the first five cycles (as shown in Figure 5b) also suggests that the surface is not stable despite that the irreversible reaction only appears to occur in the first cycle, which may explain the poor cycling performance of the pristine LTO. The hydrothermal‐treated LTO with water shows another pair of redox peaks at 1.70 and 2.08 V (Figure 5c,d), attributed to lithium intercalation and deintercalation concerning the anatase‐TiO_2_ phase.^38^ While for the modified LTO by Na_2_S‐assisted hydrothermal treatment (Figure 5e,f), no additional current peaks were observed, implying that the high‐quality surface possesses better electrochemical properties. Thus, the surface–property relationship is revealed by the detailed CV analysis, supporting that the high‐quality surface, without the amorphous or anatase‐TiO_2_ surface layer, is responsible for the improved electrochemical performance.

## 3 Conclusions

In summary, a facile modification method was introduced for Li_4_Ti_5_O_12_ anode materials existing of simple hydrothermal treatment with a Na_2_S aqueous solution. Based on the HR‐TEM, XPS, and Raman observations during the treatment, an ≈10 nm amorphous layer at the outmost layer of pristine Li_4_Ti_5_O_12_ was entirely removed resulting in clean and nondefective surfaces. The subsequent electrochemical measurements show that the modified Li_4_Ti_5_O_12_ spheres possess a higher Coulombic efficiency (94.9%) during the first cycle, a longer cycle life, and a better rate capability. It is interesting that the hydrothermal treatment with water results in anatase‐TiO_2_ on the Li_4_Ti_5_O_12_ surface, while the hydrothermal‐treated Li_4_Ti_5_O_12_ sample with the Na_2_S aqueous solution maintains a pure and high‐quality spinel‐Li_4_Ti_5_O_12_ phase. The surface reconstruction mechanism and chemical process were carefully investigated through a series of experiments with different concentration of Na_2_S aqueous solution and hydrothermal treatment time. The combined Raman and ICP analysis revealed that the interaction of water and Li_4_Ti_5_O_12_ particle is a hydrolysis‐recrystallization process, while with a proper amount of Na_2_S (0.004 mol L^−1^ at least), the recrystallization process can be totally hindered due to the adsorption of S^2−^ or HS^−^ ions. These findings shed new light on the importance of surface phenomena that need to be fully understood and ultimately controlled by developing practical methods for electrode material preparation and modification.

## 4 Experimental Section

*Surface‐Modified Method*: The LTO material used in this study was purchased from Ishihara Sangyo Kaisha Co. Ltd. (Japan). The LTO particles were modified using a simple hydrothermal procedure. Typically, 5 g of LTO and 0.16 g of Na_2_S•9H_2_O (Aldrich) were thoroughly mixed in 80 mL of water (i.e., ≈0.008 mol L^−1^ Na_2_S solution) at ambient temperature. Then the solution was transferred to a 100 mL Teflon‐lined stainless reactor and placed in an oven at 180 °C for 8 h. After that, the solution was centrifuged, collected, and taken to detect the concentration of soluble lithium and titanium by ICP tests (Agilent). Meanwhile, the white solid samples were collected by direct filtrating, washing with water, and then drying at 120 °C. To investigate the surface reconstruction process and mechanism, a series of analogous experiments were performed with 5 g LTO and different concentrations of Na_2_S (from 0 to 0.02 mol L^−1^) and different hydrothermal duration (2, 4, 8, and 12 h).

*Structural Characterization*: The morphology and size of the LTO were characterized using a HITACHI S4800 scanning electron microscope (SEM). HR‐TEM was performed using a 300 kV field emission‐TEM (FE‐TEM; Tecnai G^2^F30; FEI, USA). The phase and the crystallographic structure of the LTO were characterized by powder X‐ray diffraction using Cu Kα radiation (λ = 1.5418 Å) at the range of 10°–70° (XRD; D/Max 250/PC; Regaku, Japan). Synchrotron X‐ray was also employed to detect the possible minor phase of anatase‐TiO2. The X‐ray diffraction data were obtained at beamline BL14B1 of the Shanghai Synchrotron Radiation Facility (SSRF) using X‐ray with a wavelength of 0.6896 Å. The detailed information about beamline BL14B1 can be found in ref. 39. The surface structures of LTO were characterized by Raman spectroscopy employing an excitation wavelength of 532 nm at room temperature (LabRam HR800; HORIBA Jobin Yvon Co. Ltd., France). XPS analyses were conducted with a spectrometer using focused monochromatized Al Kα radiation (Thermo Fisher, ESCALAB 250Xi, USA).

*Electrochemical Characterization*: Electrochemical tests were carried out using coin‐type cells (CR2032) assembled in an argon‐filled glove box. The working electrode was fabricated by casting the mixture of active material (LTO; 80 wt%), super‐P (SP; 10 wt%), and poly(vinyl difluoride) (PVDF; 10 wt%) onto an aluminum foil. A polypropylene film (Celgard 2300; USA) was used as a separator, and the pure metallic lithium foil was used as a counter electrode. The 1 m LiPF_6_ in ethylene carbonate (EC)/dimethyl carbonate (DMC)/diethyl carbonate (DEC) (1:1:1) solvent was employed as electrolyte. In our study, the loading mass of active materials is about 7.2 mg cm^−2^. The cycling and rate capability measurements of the assembled cells were carried out using a Land CT2001A system. The CV tests were performed using an electrochemical workstation (Solartron 1470E, UK). All the described cell tests were performed in the voltage range of 1.0−2.5 V (vs Li^+^/Li) at 25 °C.

## Conflict of Interest

The authors declare no conflict of interest.

## Supporting information

Supplementary
