# Supplementary material for: A Facile Surface Reconstruction Mechanism toward Better Electrochemical Performance of Li4Ti5O12 in Lithium‐Ion Battery
Source: Adv Sci (Weinh). 2017 Jul 10;4(11):1700205. doi: 10.1002/advs.201700205 (PMC5700637; doi:10.1002/advs.201700205)
Supplement: Supplementary file 1 — Supplementary [file ADVS-4-na-s001.pdf]

Copyright WILEY-VCH Verlag GmbH & Co. KGaA, 69469 Weinheim, Germany, 2017.

ADVANCED SCIENCE
Open Access

Supporting Information

for *Adv. Sci.,* DOI: 10.1002/advs.201700205

A Facile Surface Reconstruction Mechanism toward Better Electrochemical Performance of $Li_4Ti_5O_{12}$ in Lithium-Ion Battery

Kun Qian, Linkai Tang, Marnix Wagemaker, Yan-Bing He, Dongqing Liu, Hai Li, Ruiying Shi, Baohua Li, and Feiyu Kang**

Copyright WILEY-VCH Verlag GmbH & Co. KGaA, 69469 Weinheim, Germany, 2016.

Supporting Information

A facile surface reconstruction mechanism towards better electrochemical performance of $Li_4Ti_5O_{12}$ in lithium ion battery

Kun Qian, Linkai Tang, Marnix Wagemaker, Yan-Bing He, Dongqing Liu, Hai Li, Ruiying Shi, Baohua Li,* and Feiyu Kang*

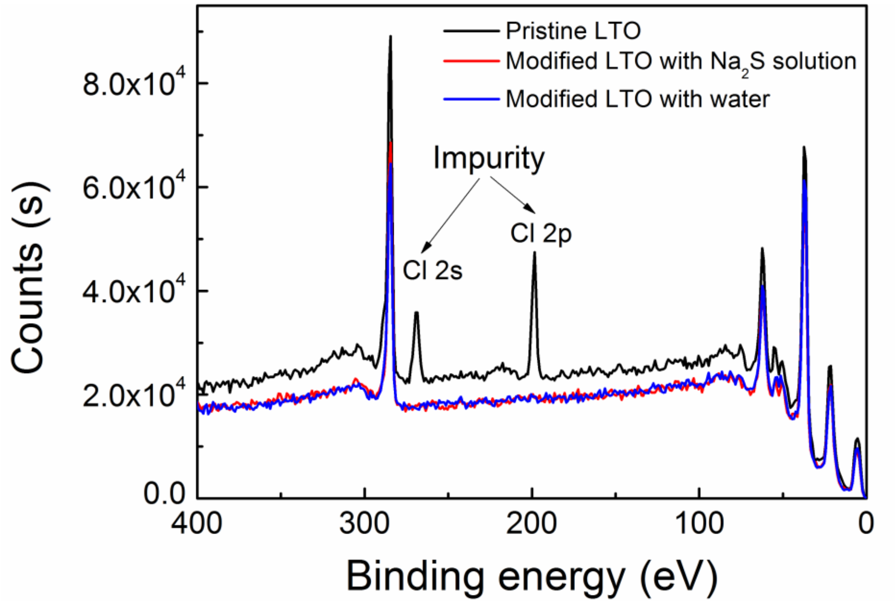

Figure S1. XPS spectra of pristine $Li_4Ti_5O_{12}$, water treated $Li_4Ti_5O_{12}$ and Na_2S treated $Li_4Ti_5O_{12}$.

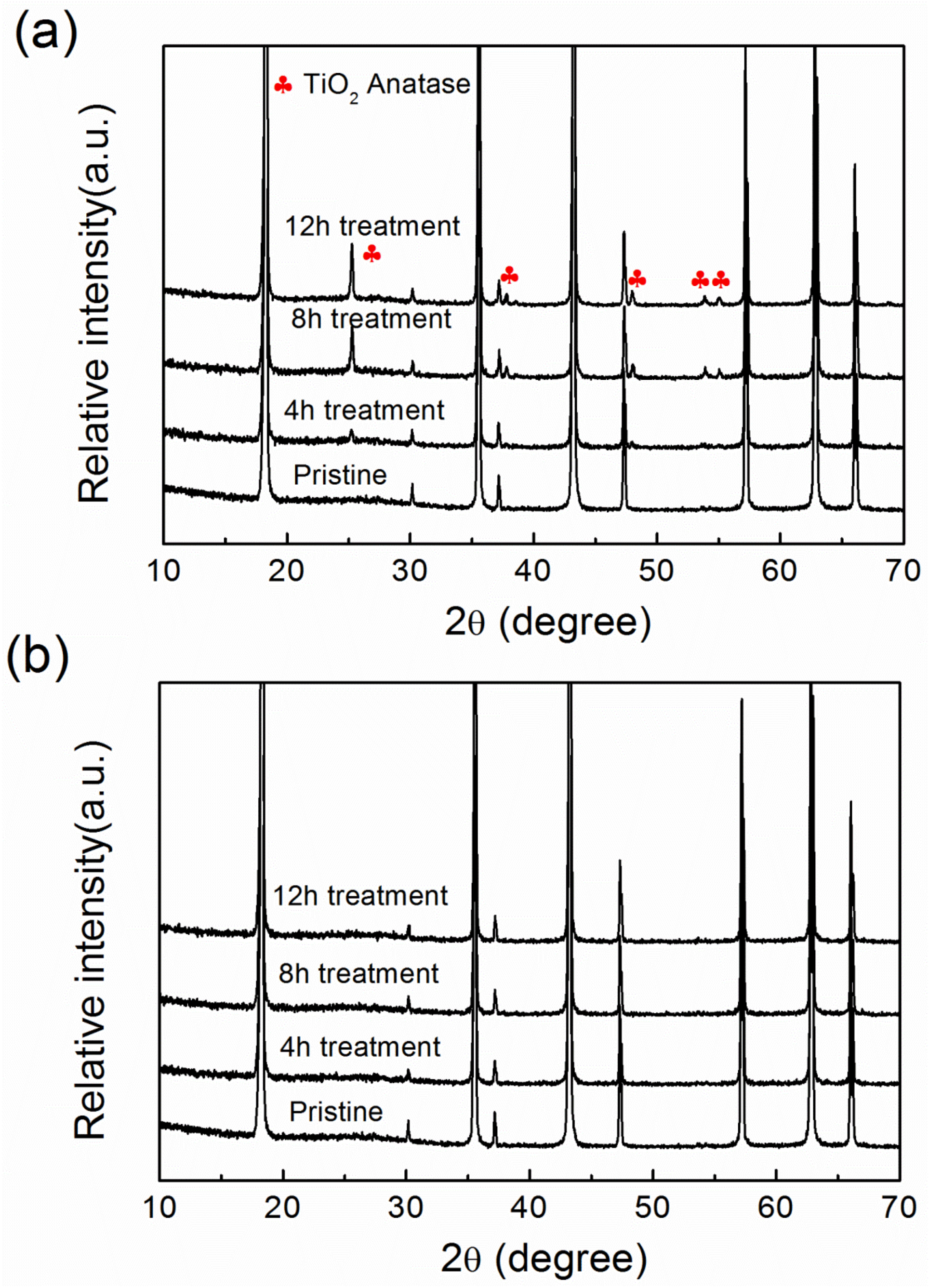

Figure S2. XRD pattern of (a) water treated $Li_4Ti_5O_{12}$ and (b) Na_2S solution treated $Li_4Ti_5O_{12}$ with different hydrothermal duration.

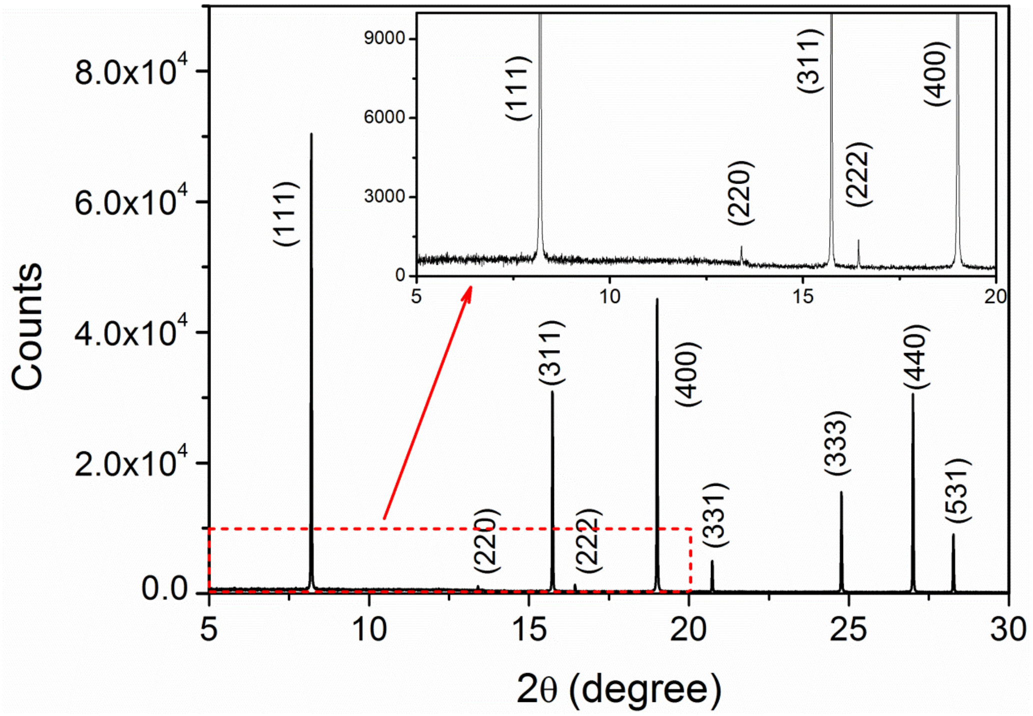

Figure S3. Synchrotron XRD pattern of $Li_4Ti_5O_{12}$ after 12 h Na_2S solution treatment.

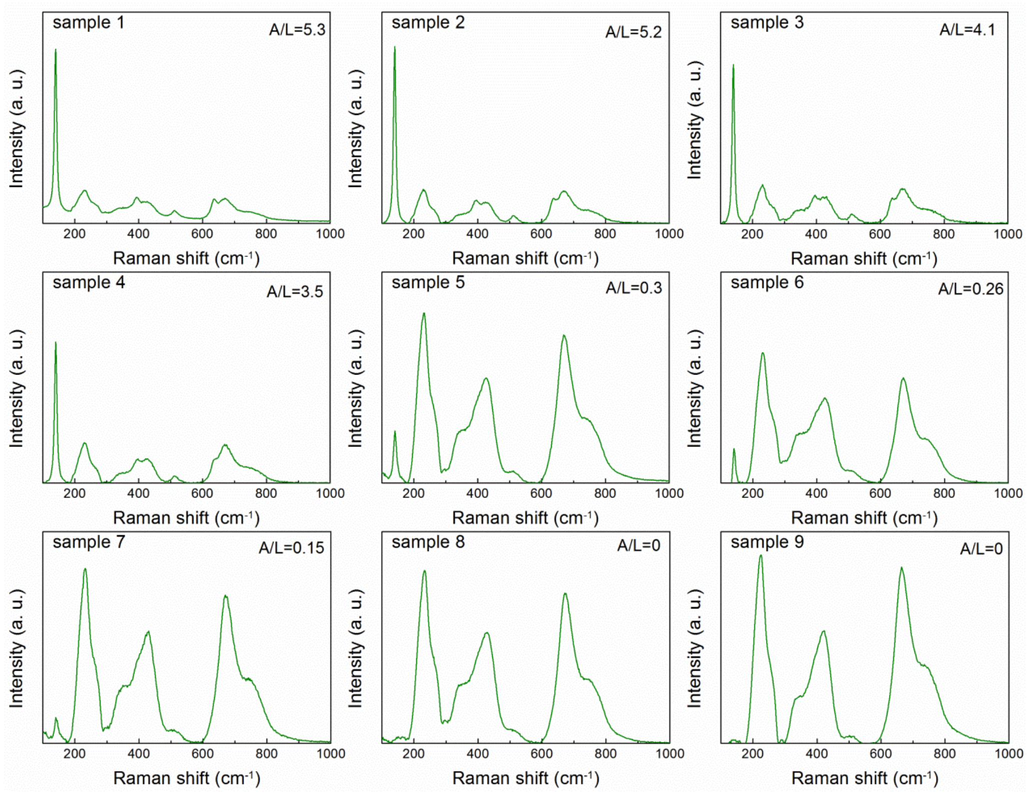

Figure S4. Raman spectra of 8h-hydrothermal treated $Li_4Ti_5O_{12}$ with different concentrations of Na_2S aqueous solution.

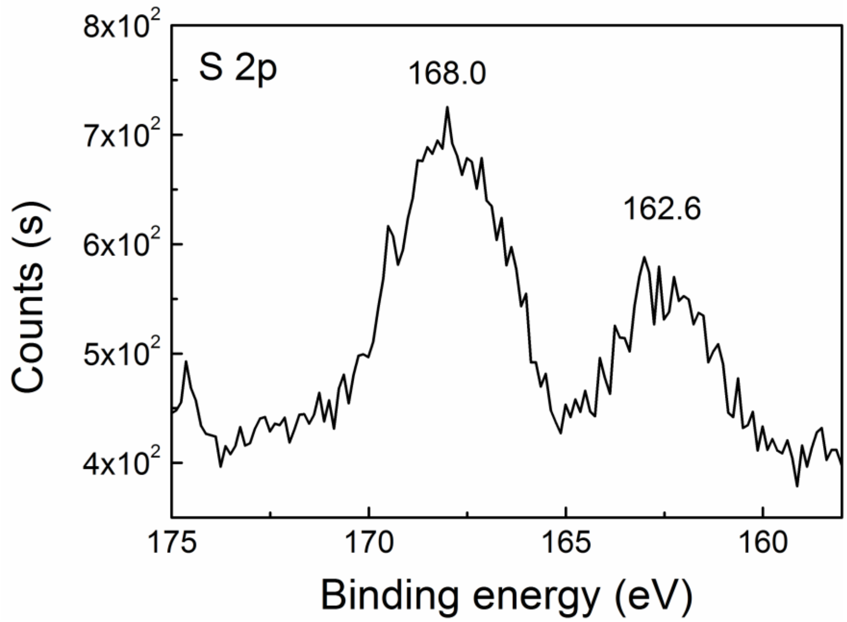

Figure S5 Narrow XPS spectrum of S 2p in Na_2S treated LTO (washed there times). The sulfur exists on the surface of Na_2S treated LTO. The peak at 162.6 eV is attributed to the HS^-/S^{2-} (NIST XPS data base) while the peak at 168 eV may come from the S in the LTO lattice. This result clearly confirm that the presence of sulfur on the LTO surface which is in accord with our inference.

Table S1. Conditions sample 1-10 to study surface modification mechanisms of LTO particles and their corresponding A/L value.

	Sample1	Sample2	Sample3	Sample4	Sample5	Sample6	Sample7	Sample8	Sample9	Sample10
Na_2S Concentration (mol L^{-1})	0	0.0012	0.002	0.0024	0.0028	0.0032	0.0036	0.004	0.008	0.02
A/L value	5.3	5.2	4.1	3.5	0.3	0.26	0.15	0	0	0

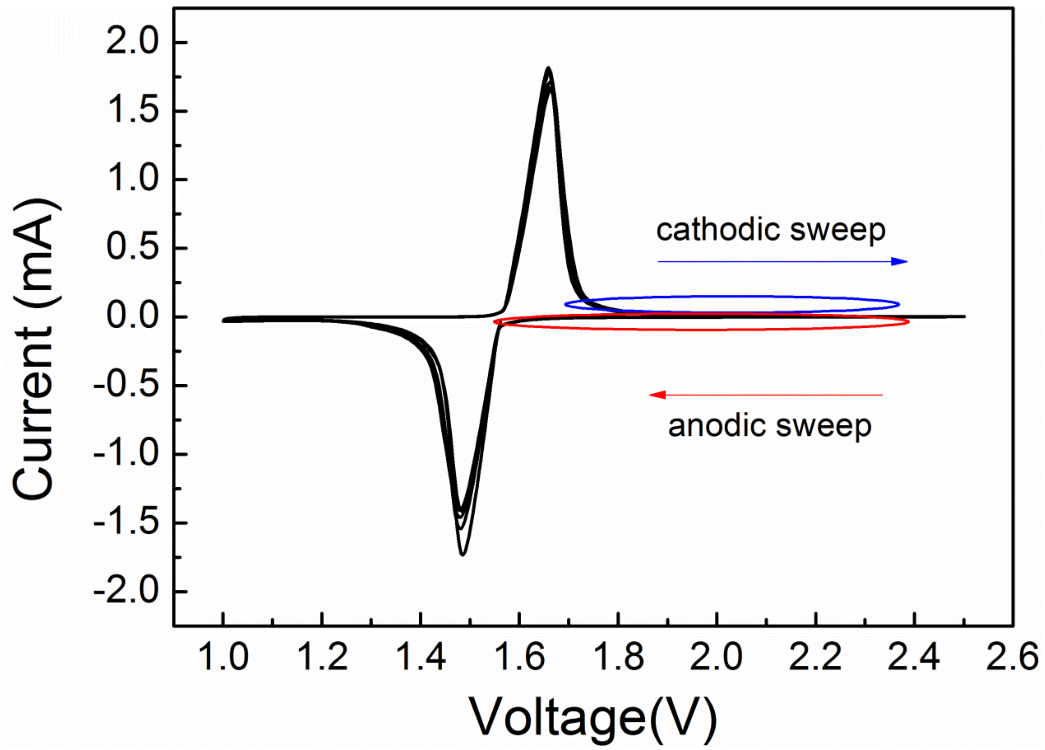

Figure S6. Typical cyclic voltammetry curves of $Li_4Ti_5O_{12}$ at a scan rate of 0.1 mV s^{-1}.
